# Postprandial hypotension is more common than orthostatic hypotension in older adults with dementia with lewy bodies: a cross-sectional study

**DOI:** 10.1038/s41440-024-01829-x

**Published:** 2024-08-13

**Authors:** Ahmet Turan Isik, Mehmet Selman Ontan, Fatma Sena Dost, Feyza Mutlay, Alev Cam Mahser, Acelya Gokdeniz Yildirim, Derya Kaya

**Affiliations:** 1 Unit for Aging Brain and Dementia, Department of Geriatric Medicine, Faculty of Medicine, Dokuz Eylul University, Izmir, Turkey; 2 Kocaeli Darıca Farabi Training and Research Hospital, Department of Geriatrics, Kocaeli, Turkey

**Keywords:** Dementia with Lewy Bodies, Postprandial Hypotension, Orthostatic Hypotension

## Abstract

Cardiovascular autonomic dysfunction is one of the supportive clinical features in dementia with Lewy bodies (DLB). This study aimed to investigate the frequency of postural and postprandial hypotension in people with DLB. The study group comprised 125 patients with DLB (76 females; mean age 78.4 ± 7.1 years) and 122 controls (88 females; mean age 74.4 ± 6.9 years). Postprandial blood pressure changes were assessed by ambulatory 24-hour blood pressure monitorization. Postural blood pressure changes were assessed via the head-up tilt table test. The frequency of postprandial hypotension (PPH) and orthostatic hypotension (OH) was higher in patients with DLB compared to controls (89.4% vs 51.7%; *p* < 0.001, and 45.5% vs 27.9%; *p* = 0.004, respectively) whereas the frequency of supine hypertension (SH), and orthostatic hypertension (OHT) was similar. However, SH in non-hypertensive participants was higher in DLB patients than in controls (48.9%, 25.7%; *p* = 0.035). PPH and OH were independently associated with a diagnosis of DLB (odds ratio [OR]:10.26 confidence interval [CI]%95 3.02–34.82; *p* < 0.001, and OR:2.22 CI%95 1.2–4.12; *p* = 0.012, respectively) after adjustment for age, number of medications, use of anti-psychotics drugs, angiotensin receptor blockers, and beta blockers. In conclusion, the study demonstrated that PPH was the most common finding of cardiovascular autonomic dysfunction, followed by OH and SH in older patients with DLB. Given the potential complications of postural blood pressure changes and PPH in such patients, cardiovascular autonomic dysfunction should be evaluated in patients with DLB.

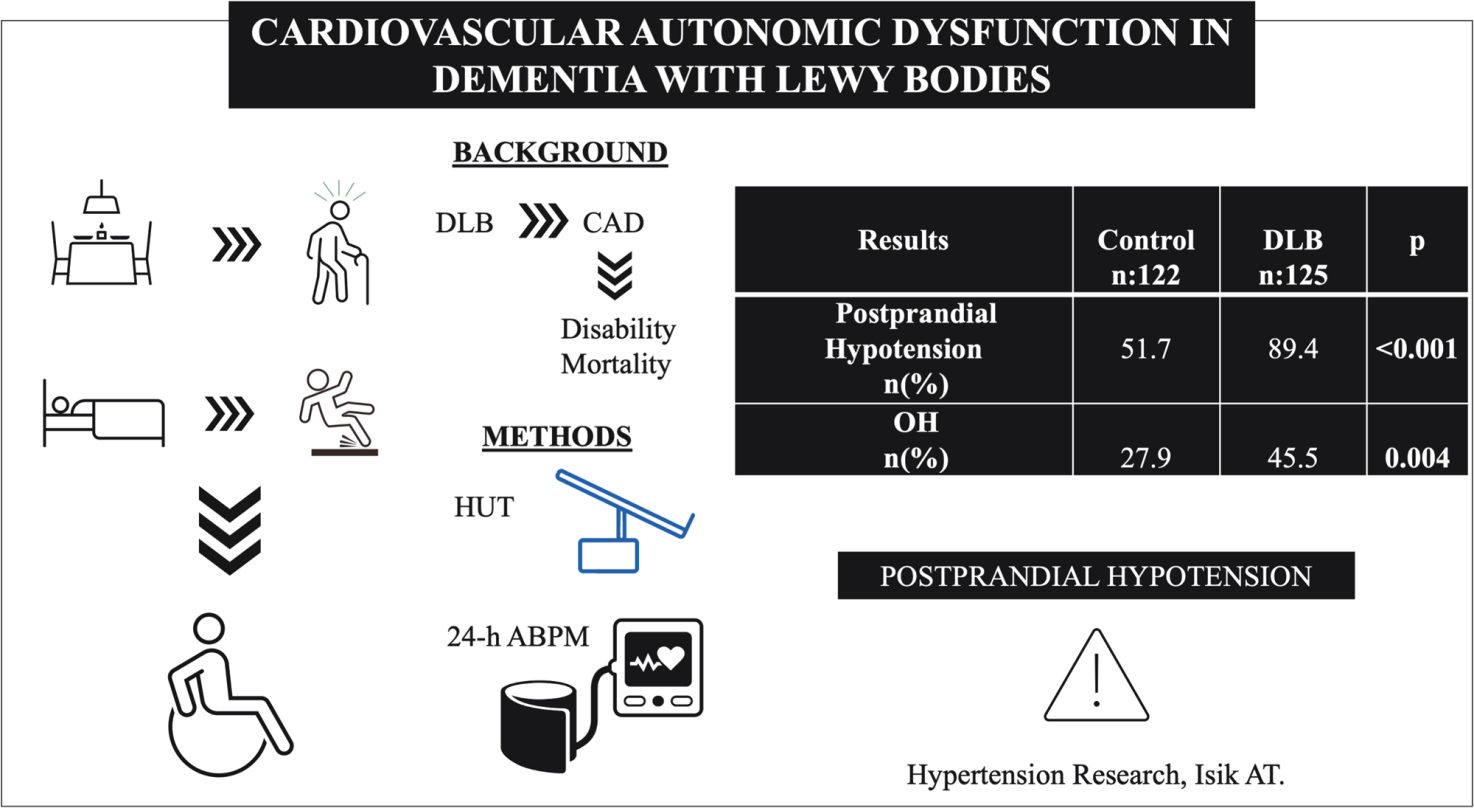

## Introduction

Dysautonomia, a dysfunction of the autonomic nervous system (ANS) controlling many fundamental homeostatic mechanisms within our physiology, such as heart rate, blood pressure, body temperature, salivation, pupil size, gastrointestinal motility, and sphincter control, may accompany many disease processes [1]. There is growing evidence in the literature that dysautonomia, occurring at all the stages of the synucleinopathies, such as Parkinson Disease (PD), Dementia with Lewy Bodies (DLB), and Multiple-System Atrophy, may be an initial clinical finding of the early disease stage [2]. A diagnosis of dementia is associated with an increased risk of orthostatic hypotension [3], and this association is the strongest in DLB [4, 5]. Dysautonomia, including urinary incontinence, OH, and constipation, is suggested to be an early and prominent marker of DLB [6–9], one of the most common clinical syndromes of synucleinopathies [10].

The hallmark of the pathology is accounted for by the aggregation of α-synuclein in neurons and glia, identified as Lewy bodies found in the post-synaptic peripheral autonomic neurons. DLB is also associated with under activity of the cholinergic nervous system [6], including parasympathetic and pre-ganglionic sympathetic neurotransmission [11]. In addition, neuronal loss in DLB is variable in most cases, commonly ensuing in the nigrostriatal dopaminergic pathway, olfactory bulb, locus coeruleus, amygdala, limbic and superior temporal cortices, and peripheral nervous system [10]. Clinical manifestations are varied including parkinsonism, fluctuating cognition, dementia, visual hallucinations, REM sleep behavior disorder (RBD) and autonomic dysfunction.

Neurogenic OH, a classic manifestation of cardiovascular autonomic dysfunction, is associated with cognitive impairment, incident falls, and reduced survival in patients diagnosed with DLB [12]. It is not only one of the supportive clinical features of the DLB but also leads to complexity in the management of patients and increases disease burden [13]. Although OH has been investigated in DLB [14], other manifestations of cardiovascular autonomic dysfunction such as postprandial hypotension (PPH), orthostatic hypertension (OHT) and supine hypertension (SH), in such patients have not been studied in detail. Therefore, this study aimed to investigate the frequency of OH, PPH, OHT, and SH in patients with DLB.

## Methods

### Participants

Between December 2017 and April 2023, a total of 125 patients with probable DLB were included in the study. One hundred twenty-two cognitively healthy individuals who were admitted to the geriatric clinic for evaluation of forgetfulness but with normal cognitive assessment and normal physical and neurological examinations served as controls. The diagnosis of probable DLB was made according to the fourth consortium of DLB consensus [8], which was confirmed by DaT-Scan Imaging in 10 patients for whom the procedure could be performed. The study was approved by the local ethics committee of the University and was conducted in accordance with the Helsinki Declaration.

All the patients underwent comprehensive geriatric assessment (CGA), including a complete medical history evaluation, neurological and neuropsychological assessment [15, 16], and routine blood tests. Additionally, with the consent of the patients, cerebrospinal fluid (CSF) analysis was performed, and brain imaging was carried out in all the demented patients.

### Exclusion criteria

The patients with infection, peripheral neuropathy, stroke, other types of dementia than DLB, malignancy, major depression, acute or chronic inflammatory/autoimmune disease, decompensated heart failure, were excluded.

### Comprehensive Geriatric Assessment (CGA)

Data collected included demographic characteristics (age, gender), comorbid diseases, number of drugs, use of antihypertensive drugs, antipsychotics, antidepressants and antiparkinsonian medications, Barthel Index for Basic Activities of Daily Living (BADL), Lawton-Brody Instrumental Activities of Daily Living (IADL), Tinetti Performance and Mobility Assessment (POMA), brain imaging results and laboratory testing (blood, CSF) [17]. Global cognitive functions were tested with the Montreal Cognitive Assessment (MOCA) in those with 11 or more years of education and with the Mini-Mental State Examination (MMSE) in those with less than 11 years of education. Constipation was assessed by a self-report constipation questionnaire (yes/no).

### Evaluation of postural and postprandial blood pressure changes

Postural blood pressure changes were evaluated using head up tilt test (HUT, Gemesan1 Tilt TableG-71, Turkey). Monitoring throughout the HUT was conducted using the Biolight1 BIOM69. After the patients were allowed to rest in the supine position for at least 10 min in a quiet room at 20–24 °C, the Tilt Table was raised to an angle of 60–80°. The patients were monitored for blood pressure during HUT with intermittent BP monitoring at regular interval with brachial cuff [18]. OH was defined as a decrease of at least 20 mmHg in systolic blood pressure and/or 10 mmHg in diastolic blood pressure within 3 min in the HUT test in an upright position of at least 60 degrees [3]. Neurogenic OH was defined as Δheart rate/Δsystolic BP ratio < 0.5 bpm/mm Hg [19]. Delayed OH was defined as OH after 3 min during HUT [20]. OHT was defined as an increase in systolic blood pressure of 20 mmHg for normotensive individuals [21, 22]. Supine HT (SH) was defined as a systolic blood pressure of at least 140 mmHg or a diastolic blood pressure of at least 90 mmHg after staying in the supine position for at least five minutes [23]. Schiller Br-102 Plus Ambulatory Blood Pressure Monitor (Schiller AG, Baar, Switzerland) was used to record 24 h blood pressure changes in patients who could apply to it. Meal time was also recorded, and PPH was defined as a decrease in systolic blood pressure of at least 20 mm Hg within two hours of a meal [24].

### Statistical analysis

The variables were investigated using the Kolmogorov-Smirnov test to determine whether they were normally distributed. Descriptive analysis was presented using means and standard deviations (SD). Continuous variables with normal distribution were analyzed via the independent samples t-test and those with non-normal distribution via the Mann-Whitney U test. Differences between categorical variables were assessed with Pearson’s chi-square test or Fisher’s exact test. Binary logistic regression analysis was performed for the factors that were likely to affect OH, delayed OH, and PPH in patients with DLB. Adjusted model was established according to age, number of drugs, usage of antipsychotics, antiparkinsonian drugs, Angiotensin receptor blockers (ARRB), and beta-blockers. Probability (p) value of < 0.05 was considered significant. All statistical analyses were performed using the SPSS 22.0 (SPSS Inc.) package program.

## Results

### Demographics and clinical features of participants

The DLB group was older than the control group (78.42 ± 7.13 vs 74.40 ± 6.89 years, *p* < 0.001). The frequency of comorbidities was not different between the groups. The number of drugs and use of ARB and BB were higher in the control group. (*p* = 0.001, *p* = 0.046 and 0.002 respectively). Use of antipsychotics was more frequent in the DLB group compared to the control group (*p* < 0.001 and *p* < 0.001, respectively). As expected, MMSE scores, MOCA scores, POMA scores, BADL and IADL scores were higher in the control group. (*p* < 0.001). Constipation, one of the dysautonomia forms, has a higher frequency in the DLB group (22.7% vs 37.2%; *p* < 0.05). The demographic characteristics of the patients, comprehensive geriatric assessment tests, laboratory parameters, comorbid conditions and postural and postprandial blood pressure changes are summarized in Table 1.

**Table 1:** Characteristics of Participants

	Control n:122	DLB n:125	p*
**Demographics**			
Age (years)	74.40 ± 6.89	78.42 ± 7.13	**<** **0.001**
Sex - Female (%)	72.1	60.8	0.059
**Comorbidities**			
Hypertension (%)	71.3	64	0.220
Diabetes Mellitus (%)	30.3	29.9	0.944
CHF (%)	3.3	6.8	0.208
CAD (%)	27.9	21.4	0.244
CVD (%)	5.7	9.4	0.283
Hyperlipidemia (%)	15.6	18.8	0.508
Depression (%)	42.6	40.5	0.742
**Drugs**			
Number of Drugs	5.37 ± 3.83	6.59 ± 3.07	**<** **0.001**
ACEi (%)	14.8	12.8	0.656
ARB (%)	42.6	30.4	**0.046**
CCB (%)	26.2	28	0.838
BB (%)	39.3	21.6	**0.002**
Diuretics (%)	36.1	35.2	0.887
Alpha blockers (%)	7.4	4	0.251
Antidepressants (%)	36.1	48.1	0.064
Antipsychotics (%)	4.9	23.1	**<** **0.001**
**Laboratory**			
eGFR mL/min 1.73 m^2^	72.81 ± 17.58	70.36 ± 20.61	0.072
**Comprehensive Geriatric Assessment**			
MMSE	25.49 ± 3.69	16.10 ± 5.44	**<** **0.001**
MOCA	23.68 ± 3.69	17.83 ± 5.83	**<** **0.001**
POMA	25.33 ± 3.62	21.33 ± 7.26	**<** **0.001**
BADL	91.58 ± 10.43	78.45 ± 22.57	**<** **0.001**
IADL	20.04 ± 3.92	10.18 ± 6.58	**<** **0.001**
**Postural and Postprandial Blood Pressure – Pulse Changes**			
Postprandial Hypotension (%)	51.7	89.4	**<** **0.001**
Supine Hypertension (%)	37.7	46.4	0.183
OHT (%)	1.6	6.5	0.054
OH (%)	27.9	45.5	**0.004**
Neurogenic OH (%)	26.2	35.1	0.140
Orthostatic Symptom (+) (%)	10.3	11.5	0.656
Delayed OH (%)	5.7	13.3	0.054

*ACEi* Angiotension Converting Enzyme inhibitor, *ARB* Angiotensin Receptor Blocker, *BADL* Barthel Basic Activity of Daily Living, *BB* Beta Blocker, *BP* Blood Pressure (mmHg), *CAD* Coronary Artery Disease, *CCB* Calcium Channel Blocker, *CHF* Congestive Heart Failure, *DLB* Dementia with Lewy Bodies, *IADL* Lawton-Brody Instrumental Activity of Daily Living, *MMSE* Mini Mental State Examination, *MOCA* Montreal Cognitive Assessment, *OH* Orthostatic Hypotension, *OHT* Orthostatic Hypertension, *POMA* Tinetti Performance Oriented Mobility Assessment, *CVD* Cerebrovascular Disease

*Statistically significant *p*-values (*p* < 0 0.05) are shown in bold characters

### Postural and postprandial blood pressure and changes

PPH and OH, were more frequent in the DLB group (*p* < 0.001, *p* = 0.006, respectively) (Table 1). The decrease in postprandial systolic blood pressure was higher in the DLB group than control (36.88 ± 13.77, 32.82 ± 12.68 respectively). Besides, the prevalence of PPH was not different between meals. Even though there was a higher frequency of neurogenic OH in DLB group than the control (35.1% and 26.2% respectively), no significant difference was found between the groups (*p* = 0.140) (Table 1). However, when patients taking beta-blockers were excluded from both groups, neurogenic OH in the DLB group was significantly more common than in the control (38.6% vs 21.6%; *p* = 0.020). Moreover, the frequency of SH was similar in the groups (Table 1).

SH in non-hypertensive participants was more frequent in DLB patients than in controls (48.9% vs, 25.7%; *p* = 0.035). After adjustments according to age, the number of drugs, anti-psychotic, ARB (angiotensin receptor blocker) and BB (beta blocker); PPH and OH were associated with a diagnosis of DLB (OR 10.259% 95 CI 3.023–34.815 *p* < 0.001and OR 2.218**%** 95 CI 1.048–4.116 *p* = 0.012 respectively). (Table 2).

**Table 2:** The association with cardiovascular autonomic dysfunction and DLB after adjusted for covariates*

	Control	Dementia with Lewy Bodies		
	OR(95% CI)	OR	OR(95% CI)	p**
Postprandial hypotension (%)	Reference	10.259	3.023–34.815	**<** **0.001**
Orthostatic Hypotension (%)		2.218	1.195–4.116	**0.012**

*CI* Confidence Interval, *OR* Odds Ratio

*Adjusted for age, number of drugs, antipsychotic drugs, angiotensin receptor blockers, beta blockers

**Statistically significant *p*-values (*p* < 0.05) are shown in bold characters

## Discussion

This cross-sectional-single center study demonstrates that OH and PPH are more common in older patients with DLB than in controls.

When it comes to dysautonomia, which is likely to be an early marker of DLB, OH is often the first to come to mind. A recent meta-analysis has reported OH in 50.8% of DLB patients [14], which is considerably higher than both patients with AD (28%) [3] and non-demented older adults (17.9%) [5]. Many studies in patients with DLB have shown that OH may be closely associated with cognitive impairment and may even be an important predictor of cognitive outcomes, and chronic hypoperfusion may link to neurodegenerative mechanisms [4, 12, 25, 26]. Considering pathophysiological changes in OH as well as DLB, other findings of cardiovascular autonomic dysfunction such as SH, OH, delayed OH, and PPH also seem to be associated with DLB, which has not yet been evaluated in detail, especially PPH in DLB patients.

Unlike others, PPH is characterized by inadequate cardiovascular compensation to meal-induced splanchnic blood pooling resulting from complex interactions between ingested nutrients and the gastrointestinal tract [27]. Besides cardiovascular autonomic dysfunction, several potential mechanisms are involved in the development of PPH: Increased splanchnic blood pooling, age or HT-related decreased baroreflex function, inadequate sympathetic nerve firing or vascular responsiveness to norepinephrine, upregulation of vasoactive intestinal peptides, and insulin-mediated vasodilation [27]. As well as OH, PPH is also associated with an increased risk for cerebrovascular disease, transient ischemic attack, syncope, falls, new stroke, coronary events, and mortality [27, 28]. Moreover, it is reported that PPH may also be related to fatigue, presyncope, syncope and cognitive decline in demented patients [29].

The fact that half of the controls in the study had PPH confirms that older adults are prone to PPH. This may be related to an age-related selective decrease in the number of cholinergic neurons in the enteric nervous system and parallel progressive loss of Cajal interstitial cells in the stomach and colon throughout life [30]. Additionally, the very high frequency of PPH in DLB patients, nearly 9 out of 10 patients, is of paramount importance to clinical practice, indicating that an older patient with PPH should be investigated for DLB or carefully monitored for an early/upcoming feature of DLB. On the contrary, DLB patients who report worsening in their clinical condition, especially after meals, should be evaluated for PPH, and all the aforementioned adverse health outcomes related to PPH should be prevented in DLB patients.

This study showed that DLB increased the risk of OH and PPH by approximately 2 and 10-fold, respectively, compared to controls consistent with our previous studies [8, 9, 14]. On the other hand, despite the higher frequency of OH in the DLB group than in the controls, the similarity of the frequency of orthostatic symptoms between the groups suggests that the ability to recognize the symptoms may be impaired in patients with DLB [31]. Although it is known that the frequency of neurogenic OH is especially increased in DLB [32], no significant difference was detected between the control group and the DLB group (26.2% vs 35.1% respectively) in our study, which seems to be due to the widespread use of beta-blockers in the control group. (39.3% (control group) vs 21.6% (DLB group)). In other words, the negative chronotropic effect of the drugs may prevent increased the heart rate in the patients. Accordingly, when patients receiving beta-blockers were excluded from both groups in this study, we found that the frequency of neurogenic OH in the DLB group was higher than in the control (38.6% vs 21.6%).

To the best of our knowledge, this is the one of the first studies to report the increased risk of PPH in DLB patients. Considering both the adverse health outcomes associated with DLB [9, 13, 19, 33], and adverse events and complications related to OH and PPH, as signs of cardiovascular autonomic dysfunctions, it is clear that the prevention of such outcomes is indispensable to ensure better management of DLB [8, 9, 14]. For this purpose, physicians may consider some non-pharmacological interventions as well as some pharmacological interventions in order to treat patients with PPH. Among them, non-pharmacological interventions are i) eating smaller meals more frequently and drinking more water, ii) ten minutes of postprandial walking nearly 20 minutes after breakfast, and iii) cold glucose loading, while the other includes i) caffeine, an adenosine receptor antagonist, ii) somatostatin analogs for local vasoconstriction to reduce splanchnic blood flow, iii) acarbose which is an alpha-glucosidase inhibitor, and iv) calcitonin gene-related peptide inhibitors to inhibit vasodilatory activity in response to glucose loading [34–38].

The frequency of delayed OH in DLB patients was similar to that in the controls. Indeed, a higher frequency is an expected outcome, as delayed OH is likely to be an early and milder form of OH in such patients. Within this context, Gibbons et al. reported that delayed OH seems to be associated with progression to OH and 10-year mortality in patients with α-synucleinopathies [14, 20].

Additionally, in the DLB group, the frequency of SH, one of findings of the cardiac autonomic dysfunction that can be impacted by age and renal function [39], was similar to that in the controls. However, when SH was evaluated according to the presence of HT in each group in order to eliminate the effect of HT, it was shown that SH was higher in DLB patients without HT than in those of controls.

Moreover, the frequency of OHT, another and probably the least investigated finding of postural blood pressure change, was similar in both groups, the pathophysiology of which is not fully elucidated and requires systematic evaluation. Given the adverse health outcomes associated with DLB, as well as the fact that cardiovascular autonomic dysfunctions such as OH, SH, and PPH alone cause a variety of serious adverse health outcomes in older adults, including cardiovascular events, cerebrovascular disease, syncope, falls, fractures, cognitive impairment and mortality [3, 5, 14, 40], the prevention of such conditions is crucial to ensure better management of DLB and alleviate caregiver burden.

The study has several strengths. To start with, all the participants underwent a comprehensive geriatric assessment, and CSF biomarkers, if possible, were used to support the diagnosis. In addition, as far as we are concerned, this is the first study to evaluate blood pressure changes as an indication of cardiovascular autonomic dysfunction, especially PPH, in older patients with DLB. Finally, the blood pressure changes were assessed by HUT with 24-hour ambulatory blood pressure measurement and adequate sample size. On the other hand, the study has some limitations: One of which is that the study’s cross-sectional design limits the establishment of a cause-and-effect relationship. The fact that controls were unmatched to DLB cases according to age and that postural blood pressure changes cannot be evaluated by beat-to-beat monitorization in HUT may be among other limitations. Furthermore, because most of patients with HT were treated with various combination of antihypertensive medications, we could have no chance to evaluate effects of each medication group on PPH in our study population. Besides, although the diagnosis of PPH without a standard meal seems to be a limitation, 24-hour blood pressure monitoring along with the patient’s routine daily diet to diagnose PPH may be more important in terms of reflecting real-life naturalistic data. As cardiovascular medications are vital in older adults, it is considered unethical to discontinue them [41]. All the patients continued to take the medications during the tests, which was taken into account in the regression analysis. The other is the diagnosis of DLB, which is not confirmed neuropathologically.

In conclusion, this study shows that symptoms of cardiovascular autonomic dysfunction, such as OH, SH, and PPH, appear higher in DLB patients than controls. Therefore, considering the potential complications of postural blood pressure changes and PPH, it would be appropriate to evaluate cardiovascular autonomic dysfunction in the follow-up of DLB for which there is no cure yet.
